# Microstructural and Textural Investigation of an Mg-Zn-Al-Ca Alloy after Hot Plane Strain Compression

**DOI:** 10.3390/ma15217499

**Published:** 2022-10-26

**Authors:** Kristina Kittner, Madlen Ullmann, Ulrich Prahl

**Affiliations:** Institute of Metal Forming, Technische Universität Bergakademie Freiberg, Bernhard-von-Cotta-Straße 4, 09599 Freiberg, Germany

**Keywords:** dynamic recrystallization, plane strain compression, Mg-Zn-Al-Ca alloy, texture, twinning

## Abstract

The formability of magnesium alloys can be significantly improved by Ca as an alloying addition. Compared to conventional alloy sheets such as AZ31, texture modification can be found in rolled Mg-Ca sheets, which reveal a randomized orientation distribution. The hot deformation behavior of a twin-roll cast and homogenized Mg-2Zn-1Al-0.3Ca (ZAX210) alloy was characterized during hot compression at a temperature of 350 °C and strain rates of 0.1 s^−1^ and 10 s^−1^. Electron backscatter diffraction (EBSD) analysis was performed in order to describe the microstructural and texture evolution. The ZAX210 alloy exhibits a pronounced dynamic recrystallization (DRX) behavior during compression at high strain rates, while at lower strain rates DRX hardly occurred. This effect can be attributed to different DRX mechanisms that take place as a function of strain rate. At low strain rates, DRX occurred locally at the grain boundaries of the original microstructure, forming a so-called necklace structure. Increasing strain rate results in an increased fraction of recrystallized grains from 18% (0.1 s^−1^) to 39% (10 s^−1^). The microstructure revealed that twin boundaries act as nucleation sites for the DRX (TDRX). The recrystallized areas exhibit a weaker texture compared to the deformed microstructure.

## 1. Introduction

Magnesium alloys containing calcium have been focused on by the research in recent years due to their beneficial effect on grain refinement [1], creep resistance [2] and high temperature properties [3,4]. At the same time, Ca enables the adjustment of high strengths in adapted alloy systems in the process of thermomechanical treatment, for example, in Mg-Al [3], Mg-Mn [5], Mg-Al-Mn [6], Mg-Zn-Mn [7,8] and Mg-Al-Zn [9,10,11]. Further advantages in the combination of Zn and Ca in Mg alloys are the weakening of the basal texture and the improvement of formability. Consequently, various novel alloys have already been developed and investigated: Mg-2.4Zn-0.1Ag-0.1Ca-0.1Zr [12], Mg-3Zn-0.3Zr-0.25Ca-0.15Mn [13], Mg-1Sn-1Ca-0.5Zn [14] and Mg-2Zn-1Al-0.3Ca [15,16,17,18,19,20]. The investigations are mainly aimed at microstructure and texture development, considering the mechanical property profile and formability as a result of thermomechanical treatment. The mechanisms responsible for the change in texture and the influence on properties have been poorly investigated to date and are consequently not fully understood.

Alloys of the Mg-Zn-Al-Ca (ZAX) system have been the subject of only few works in the last 20 years. They have been produced mainly by conventional casting methods. Rudi et al. (2000) [21] investigated the microstructure development and mechanical properties of various ZAX alloys with a Zn/Al ratio of 2:1. Semi-solid forming was carried out, whereby high strengths were achieved as a result of a fine microstructure with various intermetallic phases, in detail Mg_17_Al_12_, MgZn and Mg_32_(Al,Zn)_49_. In addition, improved high temperature properties and improved creep resistance are observed, which is attributed to the presence of the MgZn and Mg_32_(Al,Zn)_49_ phases. Comparable results are also presented by Anyanwu et al. (2000) [22] and Zhang et al. (2004) [23]. Strength and ductility depend on stacking fault energy (SFE) and the activation of several slip systems. Calcium as an alloying element is known to influence the SFE of the material and could also enhance the <c+a>-dislocation activity [24]. Therefore, the core of our own investigations on alloy development of magnesium alloys was the addition of Ca. The ZAX210 alloy was developed together with the Helmholtz Zentrum Geesthacht as part of the SubSEEMag research project. Investigations on cast and rolled material have shown that a fine-grained microstructure and a weakened texture with basal pole splitting are obtained [15,16,19]. During twin-roll casting and subsequent strip rolling, a further weakening of the basal texture can be observed. This is accompanied by an excellent mechanical and technological property profile [19].

Plastic deformation of magnesium alloys proceeds through the two main mechanisms of slip and mechanical twinning. Twinning is important when only basal slip is predominant, for example, at low deformation temperatures. Twin systems that occur in Mg materials have already been extensively described in the literature; {10$\bar{1}$2} extension twins, {10$\bar{1}$1} compression twins and {10$\bar{1}$1}〈10$\bar{1}$2〉 {1$\bar{1}$02}〈10$\bar{1}1$〉 double twins are predominantly involved in the overall forming process. Extension twins occur most frequently in hexagonal materials. Due to their low critical resolved shear stress (CRSS) of 2.4 MPa, twinning is already activated at small deformations [25,26,27]. Studies on calcium-containing Mg alloys have shown that there is a dependence between the chemical composition and the activation of individual twin modes. Kim et al. (2013) [28] found that different deformation twins are active during rolling of the alloys ZX11 and ZX61. While extension twins are preferentially found in ZX61, the ZX11 alloy exhibits a high number of double twins. This effect is attributed to the solubility of Ca in the magnesium solid solution as a function of the Zn content. Similar results are shown by Lee et al. (2014) [29] when comparing AZ31 and ZX31 after hot rolling. However, it should be noted that the formation of twins is significantly influenced not only by the chemical composition but also by the initial texture, the grain size and the deformation conditions [30,31,32,33,34,35].

If deformation twins act as starting points for recrystallization (RX), the type of twin plays a decisive role in whether and to what extent RX processes take place. Tension twins rarely serve as nucleation sites, since the matrix within the twin is unfavorably oriented for basal slip. Studies by Guan et al. (2017) [36] show that in a WE43 alloy, no RX occurs within the tensile twins during annealing. The twin boundaries are consumed during the growth phase of adjacent recrystallized grains. The authors attribute this to the low dislocation density along the twin boundaries. Rather, double twins form starting points for recrystallization. Due to basal slip, dislocations accumulate along the twin boundaries, and consequently the internal energy increases. These processes are a necessary prerequisites for RX to occur [37,38,39]. Twins can also be starting points for dynamic recrystallization (twinning-induced dynamic recrystallization, TDRX). Xu et al. (2009) [40] describe the mechanism of continuous dynamic recrystallization, starting from the formation of new grains at twin boundaries during compression of an AZ91 alloy. They assume that twinning and basal slip are active at the early stage of deformation. The resulting basal texture has a favorable orientation for the formation of compression twins, which are then easily formed at higher strains. Some works show that twin-induced dynamic recrystallization is preferential at high strain rates. Studies by Wu et al. (2010) [41] suggest that different recrystallization mechanisms are activated depending on the strain rate. At low strain rates (<1 s^−1^), nucleation in the course of DRX occurs preferentially at grain boundaries of the original microstructure. Increasing strain rates (>10 s^−1^) cause twin boundaries to serve as nucleation sites. Comparable results are presented by Yan et al. (2011) [42], Sanjari et al. (2012) [43] and Wu et al. (2014) [44]. Common to all is an increased proportion of recrystallized grains at high strain rates, which is attributed to the activation of TDRX.

Our previous investigations on a twin-roll cast ZAX210 alloy during plane strain compression also showed an increased proportion of recrystallized grains at higher strain rates [17]. Thus, the aim of the present study is to investigate the microstructural and textural evolution of the ZAX210 alloy. Plane strain compression tests were performed at 350 °C and strain rates of 0.1 s^−1^ and 10 s^−1^. Electron backscatter diffraction (EBSD) analysis was carried out. The present results are discussed based on the effect of twinning and dynamic recrystallization mechanisms on the microstructure and texture development. Finally, the responsible mechanisms of dynamic recrystallization were described for the ZAX210 alloy produced via twin-roll casting for the first time. The ZAX210 magnesium alloy is a promising alloy for use in industrial applications because of its excellent property profile and deformation behavior after hot rolling, not only at elevated but also at low temperatures [19].

## 2. Materials and Methods

The Mg-2Zn-1Al-0.3Ca alloy (ZAX210) was twin-roll cast at the pilot plant for twin-roll casting (TRC) at the Institute of Metal Forming (Technische Universität Bergakademie Freiberg). Detailed description of the TRC process, as well as of the resulting microstructure, can be found in ref. [18]. After TRC, the ZAX210 alloy was homogenized at 420 °C for 2 h.

Samples with dimensions of 20 mm (longitudinal direction = direction of twin-roll casting, DTRC), 30 mm (transverse direction, TD) and 5.25 mm (normal direction, ND corresponds to compression direction = thickness of the TRC strip) were machined from the twin-roll cast and homogenized strip. Plane strain compression tests were carried out at 350 °C under strain rates of 0.1 s^−1^ and 10 s^−1^ using a servo-hydraulic hot-working simulator. A final true strain between 0.3 and 1 corresponding to 25% and 60% of thickness reduction was achieved. Details of the plane strain compression tests are described in ref. [18]. Directly after compression, the samples were quenched into water for microstructural characterization. As a result, the microstructure that develops during the forming process as a function of the selected test conditions is retained, and recovery, recrystallization and precipitation processes are suppressed. Due to the friction between the punch and the specimen, an inhomogeneous forming degree distribution occurs as a result of the resulting flow restriction [45]. Corresponding areas were therefore selected for quantitative microstructure analysis.

The microstructure and texture of the samples were investigated in the DTRC-TD plane using EBSD. The preparation of the microstructural specimens was carried out according to Müller et al. (2004) [46]. In addition, the samples were ion polished with a voltage of 4 kV and a beam current of 2 mA for 2 h. The studies were performed with the FEI Versa 3D scanning electron microscope using a Hikari EBSD detector at the Academic Centre for Materials and Nanotechnology of the University of Science and Technology, Cracow. The accelerating voltage was between 15 and 20 kV. A step size of 0.65 µm was selected. The EDAX software was used to analyse the recorded data. The evaluation of the EBSD data, as well as the calculation of the pole figures, were performed using the MTEX Matlab Toolbox [47].

## 3. Results

### 3.1. Flow Curves

**Figure 1:**
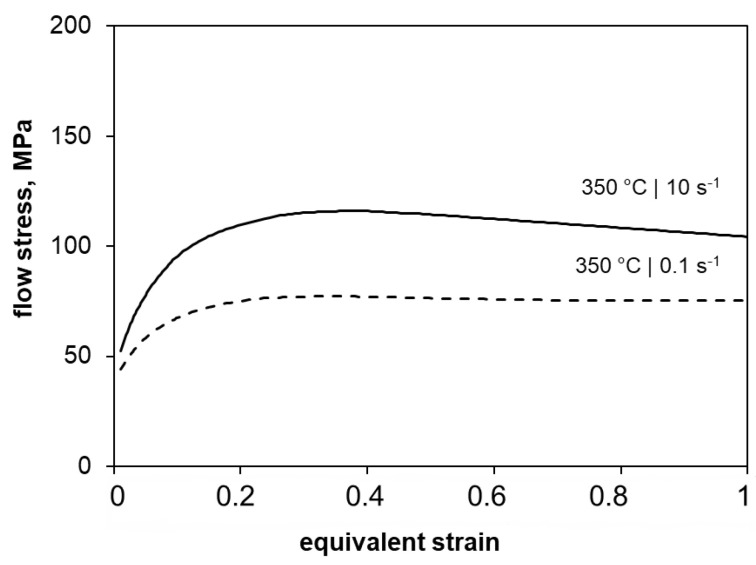
Flow curve of the twin-roll cast and homogenized ZAX210 alloy deformed by the plane strain compression test at 350 °C under a strain rate of 0.1 s^−1^ and 10 s^−1^.

Figure 1 presents the flow curves of the twin-roll cast and homogenized ZAX210 alloy deformed by the plane strain compression test at 350 °C under strain rates of 0.1 s^−1^ and 10 s^−1^. It is obvious that an increase of the strain rate results in increasing flow stresses and also shifts the flow curve maximum to higher equivalent logarithmic strains. At the beginning of the deformation, the homogenized twin-roll cast strip shows work hardening behaviour. This is accompanied by an increase in dislocation density and is characterized by a rise in the flow curve. At low strain rate, the dislocation multiplication proceeds more slowly and results in lower flow stresses. After exceeding the maximum value of the flow stress, it decreases again due to softening processes. However, no pronounced drop is visible; rather, a constant flow stress level is established. This equilibrium range is shifted toward higher flow stress values with increasing strain rate. The decrease in flow stress indicates the occurrence of dynamic recrystallization. A detailed analysis of the hot forming behaviour can be found in Kittner et al. (2019) [18].

### 3.2. Microstructural Evolution

The twin-roll cast and homogenized ZAX210 alloy exhibits a microstructure with equiaxed grains with an average cord length of 19 µm. The homogenization causes a weakening of the basal texture. At the same time, a strong spreading of the texture components along the transverse direction is evident. Detailed descriptions of the microstructure and texture of the ZAX210 alloy after twin-roll casting and subsequent homogenization can be found in ref. [18].

**Figure 2:**
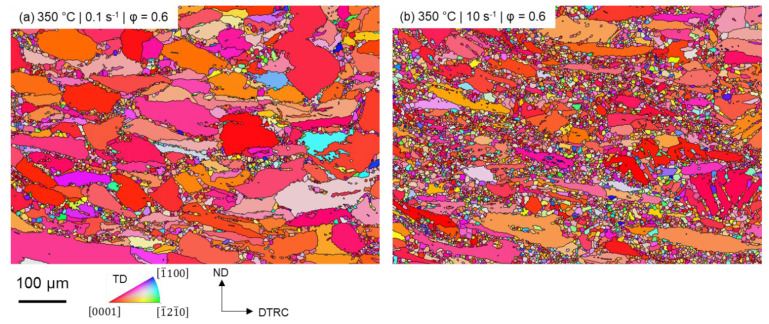
Inverse pole figure (IPF) maps of the ZAX210 alloy after plane strain compression at 350 °C under a strain rate of (**a**) 0.1 s^−1^ and (**b**) 10 s^−1^ and an equivalent logarithmic strain of 0.6.

Figure 2 shows the microstructure of the ZAX210 alloy after the plane strain compression test at 350 °C under a strain rate of 0.1 s^−1^ and 10 s^−1^ and an equivalent strain of 0.6 as an inverse pole figure. At low strain rates (Figure 2a), the original structure is predominantly deformed with only a few dynamically recrystallized grains. These form preferentially along the grain boundaries of the original structure and form a so-called necklace structure. The proportion of recrystallized grains is 18% for an equivalent logarithmic strain of 0.6 [17]. This mechanism of DRX is described for magnesium alloys by [42,43,44]. Dynamic recrystallization along the grain boundaries of the original microstructure is characterized by the newly formed grains spreading into the non-recrystallized microstructure after nucleation. This results in the formation of serrations and bulges, and finally in the formation of new grains. At the beginning of DRX, a necklace layer forms along the grain boundary. As recrystallization progresses, further layers are formed in the areas between the recrystallized microstructure and the deformed original microstructure, until the latter is finally completely consumed [48].

**Figure 3:**
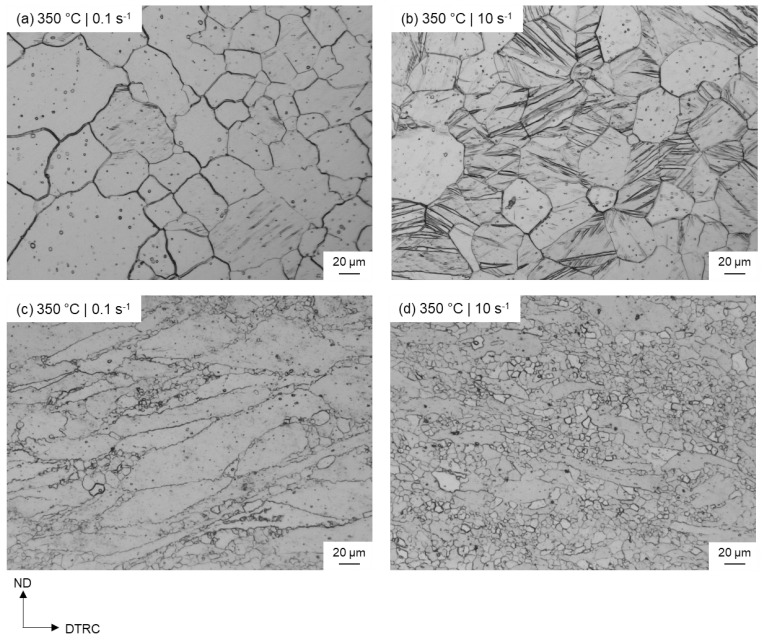
Optical micrographs of the ZAX210 alloy after plane strain compression at 350 °C under a strain rate of (**a**) 0.1 s^−1^ and (**b**) 10 s^−1^ and an equivalent logarithmic strain of 0.2, and (**c**) 0.1 s^−1^ and (**d**) 10 s^−1^ and an equivalent logarithmic strain of 0.6.

As a result of the increase in strain rate, the proportion of deformed microstructure decreases, and dynamic recrystallization is more advanced. This is also reflected in the increase of the recrystallized fraction from 18% (0.1 s^−^^1^) to 39% (10 s^−^^1^). The ZAX210 alloy shows an acceleration of the recrystallization processes (Figure 2b) with increasing strain rate and thus a special behaviour, which has currently only been described for a few magnesium alloys, especially those containing zinc [40,42,44]. Moreover, it is shown that additional dynamic recrystallization occurs at twin boundaries (TDRX). The TDRX at high strain rates and an equivalent logarithmic strain of 0.6 results in an increased fraction of recrystallized grains. This mechanism has already been found in the study of other Mg alloys in connection with increased strain rates, and is mainly initiated by the occurrence of secondary twins. In this process, new grains are formed within the twins [40,43,49]. Twinning as a forming mechanism is usually effective only at room temperature or temperatures below 200 °C [50]. However, investigations of the plane strain compression test specimens at a low logarithmic strain (φ = 0.2) show that, at high strain rates (10 s^−^^1^), a considerable amount of twinning in the microstructure compared to 0.1 s^−1^ (Figure 3) can be found. Furthermore, at higher equivalent logarithmic strain, the sample with increased twinning has a higher percentage of recrystallized grains. Whereas at low strain rates (without twinning), DRX only occurs along the original grain boundaries. The occurrence of twins at high temperatures and high strain rates is also reported by Yan et al. (2011) [42] for a ZK40 alloy and Ishikawa et al. (2005) [51] for an AZ91 alloy. They state that as the strain rate increases, the dislocation movement becomes more difficult due to lack of time and other mechanisms, such as twinning, have to accommodate the plastic deformation. Wan et al. (2020) [52] worked on enhancing the industrial-scale production of a bulk NC Mg-Gd-Y-Zr alloy by rotary swaging and concluded that high strain rates (10 s^−^^1^ to 10^2^ s^−^^1^) promote the formation of twins. Comparable results are also shown by Yang et al. (2021) [53], demonstrating that high strain rates during rotation swaging of Mg-Li alloys facilitate the activation of multiple twinning. Due to the high strain rate of 10 s^−^^1^, it is assumed that in the ZAX210 alloy, the time for dislocation climbing and gliding is limited, and twinning contributes the main part of the plastic deformation.

**Figure 4:**
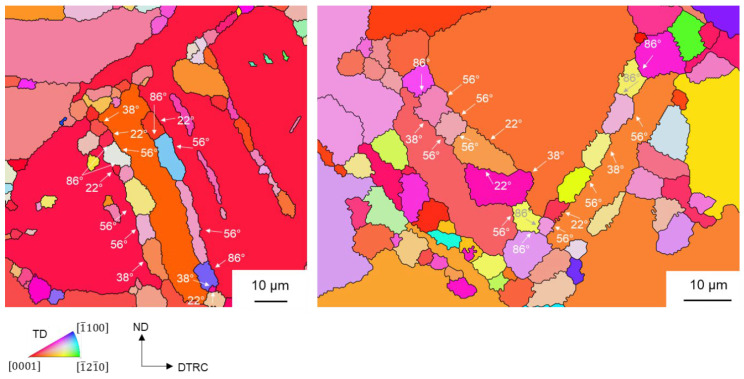
Misorientation relationships between the matrix and grains recrystallized in the twins after hot compression at an equivalent logarithmic strain of 0.6 under a strain rate of 10 s^−1^.

It is known that twins can serve as nucleation sites for dynamic recrystallization. This is especially the case when double twins occur. For this purpose, the misorientation relationships between the matrix and grains recrystallized in the twin and thus the types of twins, which occur after hot compression under a strain rate of 10 s^−^^1^, were determined from selected microstructural regions. The resulting observations are presented in Figure 4. It becomes clear that the boundaries between the twin and matrix belong to $\left\{10\bar{1}1\right\}\left\{10\bar{1}2\right\}$ (38°) and $\left\{10\bar{1}3\right\}\left\{10\bar{1}2\right\}$ (22°) double twins. At the same time, remnants of compression twins (56°) and extension twins (86°) can be identified within the compression twins. As can be seen, different twin modes exist in one grain, which is attributed to the dependence of twin nucleation on the grain boundary structure and the local stress state [54,55].

**Figure 5:**
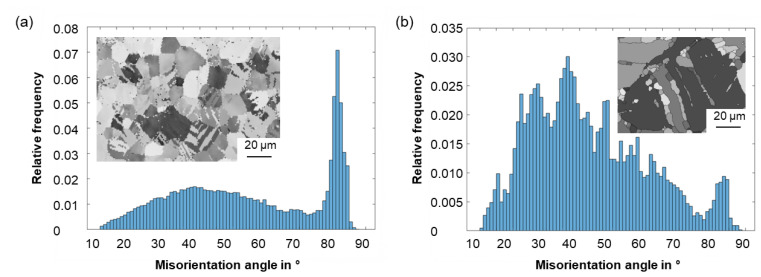
Misorientation angle distribution and electron backscatter diffraction (EBSD) map of the measured area after deformation at 350 °C under a strain rate of 10 s^−1^ (**a**) at φ = 0.2 and (**b**) φ = 0.6 (selected areas with twins).

The misorientation angle distribution of the twins occurring at high strain rates at the beginning of the deformation is presented in Figure 5a. The calculation of the twin boundaries was performed using the Brandon criterion [56]. The histogram shows a significant peak at 86°, which suggests that extension twins are formed preferentially. Based on the initial texture [17], it can be assumed that the orientation of the crystals is favorable for the activation of extension twins. As deformation proceeds, it becomes apparent that misorientation angles between 35° and 45° occur more frequently (Figure 5b). These angle ranges can be assigned to double twins. In the literature, the secondary twinning of $\left\{10\bar{1}2\right\}$-twins in primary $\left\{10\bar{1}1\right\}$-twins is described as the preferred type. These show a rotation of 37.55° about the $1\bar{2}10$-direction [57,58]. Consequently, the twin boundaries providing starting points for dynamic recrystallization are assumed to be double twins with a misorientation angle of 38°.

The mechanism of Barnett et al. (2009) [59], which involves the formation of $\left\{10\bar{1}1\right\}-\left\{10\bar{1}2\right\}$-double twins, can be used to describe dynamic recrystallization. At the beginning of the deformation, the grains of the original microstructure are separated from primary $\left\{10\bar{1}2\right\}$-extension and $\left\{10\bar{1}1\right\}$-compression twins. The formation of compression twins is favored on the one hand by a lower CRSS with increased temperature. On the other hand, the basal plane of the hexagonal cells is predominantly perpendicular to the compression direction and thus forms the preferred orientation plane for the formation of compression twins [40]. At the same time, double twins containing secondary tension twins propagate rapidly within the primary compression twins as the equivalent logarithmic strain increases. The formation of grains within the twin boundaries now takes place either by mutual crossing of the twins or the formation of double twins. Low angle grain boundaries are formed in the double twins. During the further course of the deformation process, dynamic recrystallization and finally the transition from low angle to high grain boundaries occurs. It is assumed that several mechanisms of DRX are effective during deformation of the twin-roll cast and homogenized ZAX210 alloy at high strain rates. As a result of the superposition of the formation of new grains along the grain boundaries of the original microstructure and the nucleation at twin boundaries, the recrystallization processes are accelerated, and the hot formability is improved. Particle stimulated nucleation (PSN), a mechanism often mentioned in connection with Ca-containing magnesium alloys, could not be detected. Due to the small size of the intermetallic compounds, DRX as a result of PSN was not expected [60].

### 3.3. Texture Evolution

**Figure 6:**
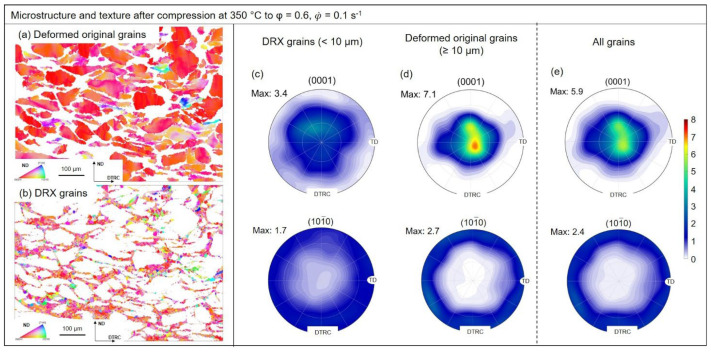
EBSD maps and pole figures of the ZAX210 alloy after hot deformation at 350 °C (equivalent logarithmic strain 0.6, strain rate 0.1 s^−1^): (**a**) EBSD map of deformed original grains, (**b**) EBSD map of DRX grains, (**c**) (0001) and $\left(10\bar{1}0\right)$ pole figures of DRX grains, (**d**) (0001) and $\left(10\bar{1}0\right)$ pole figures of deformed original grains, and (**e**) (0001) and $\left(10\bar{1}0\right)$ pole figures of all grains.

**Figure 7:**
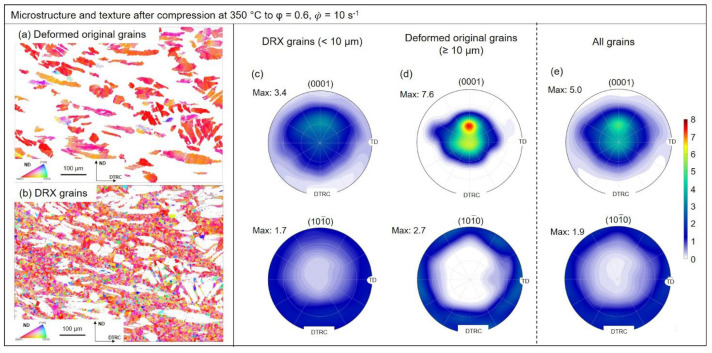
EBSD maps and pole figures of the ZAX210 alloy after hot deformation at 350 °C (equivalent logarithmic strain 0.6, strain rate 10 s^−1^): (**a**) EBSD map of deformed original grains, (**b**) EBSD map of DRX grains, (**c**) (0001) and $\left(10\bar{1}0\right)$ pole figures of DRX grains, (**d**) (0001) and $\left(10\bar{1}0\right)$ pole figures of deformed original grains, and (**e**) (0001) and $\left(10\bar{1}0\right)$ pole figures of all grains.

EBSD maps and pole figures from Figure 6 and Figure 7 were used to evaluate texture evolution during hot deformation of the ZAX210 magnesium alloy at 350 °C. The analysis was performed at an equivalent logarithmic strain of 0.6 for strain rates of 0.1 s^−^^1^ and 10 s^−^^1^. For the evaluation, a subdivision into recrystallized grains and deformed original microstructure was applied. The formation of substructures during deformation complicates the differentiation between the individual microstructural constituents. The selection of the recrystallized grains was therefore based on two essential criteria: (1) the misorientation within the grain must not exceed 2°, and (2) the recrystallized grain must be surrounded by high angle grain boundaries (>15°). Comparable criteria were also used by Humphreys et al. (2004) [61].

The results of the Investigations on texture evolution during deformation allow the conclusion that the recrystallization processes taking place cause changes of the resulting texture. Comparison of the deformed and recrystallized microstructure reveals that the deformed original microstructure exhibits a basal texture with a broadening in the transverse direction (TD), regardless of the strain rate. In addition, the intensity maximum is slightly displaced in the TRC-direction away from the core intensity. The maximum intensities of 7.1 (0.1 s^−^^1^) and 7.6 (10 s^−^^1^) indicate a strongly pronounced basal texture, in which the predominant number of basal planes of the crystallites are aligned parallel to the sheet plane. These textures arise when the two competing deformation mechanisms of basal slip and <c+a>-slip occur simultaneously. The six-fold symmetry in the prismatic pole figure normally occurs after deformation, when prismatic slip plays a strong role [62]. However, the intensity is less pronounced, so it is assumed that the of prismatic sliding is low. The newly recrystallized grains have a weakened texture (maximum intensity 3.4). Although the texture has a basal character, a clear broadening of the basal pole can be observed in both the TRC and TD directions. In addition, the intensity maxima are shifted more strongly in the TRC-direction. Bhattacharjee et al. (2014) [63] show that similar textures are obtained for recrystallized grains and non-recrystallized microstructural regions after rolling and heat treating a ZK60 alloy. The formation of grains with new, random orientations, different from those of the original microstructure, are formed as a result of various recrystallization mechanisms during deformation, and is reported in Basu et al. (2014) [64] or Griffith et al. (2015) [65]. Potential mechanisms responsible for the weakening of textures include PSN, recrystallization at deformation and shear bands, the change in grain boundary mobility due to secondary phases, or recrystallization at double and compression twins [64,65,66,67]. In the present TRC and homogenized ZAX210 alloy, recrystallization due to PSN and at deformation and shear bands can be excluded. Neither suitable particles are present, nor can deformation bands be observed after deformation. The textures of the newly recrystallized grains do not differ as a function of the strain rate. This means that regardless of the recrystallization mechanism (continuous or twin-induced DRX), the recrystallized regions display a strongly weakened texture in which the c-axis of the crystallites is tilted from the normal direction (ND) predominantly to the TRC-direction. In addition, there is the possibility that Ca, due to its segregation at grain boundaries, restricts their mobility. This inhibits the preferential growth of grains and influences the intensity of the resulting texture [28].

## 4. Conclusions

In this study, microstructural and textural evolution of a twin-roll cast and homogenized Mg-2Zn-1Al-0.3Ca (ZAX210) magnesium alloy during hot compression at 350 °C was investigated. Regarding the influence of the strain rate on the dynamic recrystallization of the alloy, the following conclusions can be drawn:

- Dynamic recrystallization is the dominant process in the formation of the microstructure during hot working of the Ca-containing magnesium alloy ZAX210.
- Depending on the strain rate, different mechanisms of DRX occur. At high strain rates (10 s^−1^), the formation of secondary twins and the nucleation of new grains within the twins is predominant. At low strain rates (0.1 s^−1^), continuous DRX occurs with the formation of characteristic necklace structures.
- TDRX leads to higher amount of recrystallized grains at high strain rates.
- The recrystallized areas show a weakened texture compared to the initial state. The texture shows basal pole splitting where the maxima are tilted away from the core intensity.

With increasing proportion of recrystallized grains after deformation, it can be expected that a weak texture is obtained. This has a positive effect on the resulting properties, so that an improved formability, a good mechanical-technological property profile and a low anisotropy can be assumed.

## Data Availability

Not applicable.
